# Practical Notes

**Published:** 1909-09-25

**Authors:** 


### PRACTICAL NOTES.

**Beverages, etc., in Gout.**

Tea, cocoa, and coffee, properly prepared, are quite harmless; the greatest consumers of these know nothing of gout. Strong black coffee taken habitually after meals is not advisable. Sugar taken in moderation I regard as not only harmless, but beneficial. Many gouty persons are best without wine. But many others are the better for the use of a little good wine, taken with one meal in the day. The quantity must be well below what is commonly regarded (by men) as moderate. So-called moderate drinkers of wine, as a rule, drink far too much.—*Sir Dyce Duckworth.*

